# Anxiety and depression in the elderly due to COVID-19 pandemic: a pilot study

**DOI:** 10.1186/s43045-021-00145-1

**Published:** 2021-10-06

**Authors:** Subhash Das, Priti Arun, Ravi Rohilla, Kantadorshi Parashar, Aratrika Roy

**Affiliations:** 1 Department of Psychiatry, Government Medical College & Hospital, Chandigarh, India; 2 Department of Community Medicine, Government Medical College & Hospital, Chandigarh, India; 3 Ashadeep Mental Health Society, Guwahati, Assam India

**Keywords:** Anxiety, Comorbidity, COVID-19, Depression, Elderly

## Abstract

**Background:**

The elderly are a vulnerable section of the population who are prone to physical, mental, social, and economic deprivation. The effect of COVID-19 had a worldwide impact on all age groups, with a particularly higher mortality and morbidity rate among the elderly population. The present study was undertaken to know about the psychological morbidity in the geriatric population during the period of the COVID-19 pandemic. The study was cross-sectional and was done through a telephonic survey. Eligible elderly subjects were contacted telephonically, and the Geriatric Anxiety Scale and the Geriatric Depression Scale were administered. To evaluate the functional ability of elderly subjects, the Everyday Abilities Scale for India (EASI) scale was administered. For the telephonic survey, verbal consent was sought.

**Results:**

A total of 92 elderly subjects were included. Male outnumbered the females with a ratio of 1.8:1. Spouse and children were primary caregivers in 83.7% of the subjects. 90.2% were married, and 66.3% had earned a graduate/professional level of education. Chronic illness was present in 50% of subjects. The most common co-morbidities were hypertension (27.2%) and diabetes (21.7%). The proportion of elderly with anxiety and depression was 8.7% and 15.2% respectively.

**Conclusion:**

The elderly showed lower levels of anxiety and depression. Higher resilience among the elderly and good family support may be the reasons for such an unexpected finding. However, more studies are required to validate the findings of the current study.

## Background

As the world grapples and fights with the unprecedented novel coronavirus (COVID-19) pandemic, it has been consistently seen that the elderly population has been disproportionately impacted [1]. Due to their enervated immune systems and associated chronic underlying diseases, they are as a whole more severely affected by the infection and deaths are more common among them [2]. The global recommendations for them, therefore, include social isolation implying staying indoors and desisting contact with others for several months in a row now [3]. Given the physical, affective, and cognitive complexities that come at this vulnerable age, a pandemic like the one we are living through can exacerbate existing challenges to well-being and also create new ones. It is well known that anxiety and depression are quite prevalent in the elderly. The pandemic’s scope and drawn-out nature are indicative that lessons learned from managing natural disasters in the past may not be entirely pertinent, and yet one cannot deny the elderly people’s significant contributions in disaster preparedness and response, which holds up that they may have important lessons for COVID-19 sufferers as well as healthcare workers across all age groups [4].

In India, the elderly population constitutes 8.6% of the total population and it is projected to reach 19% by the year 2050 [5]. As of now, the globally Indian subcontinent is the second-worst affected in terms of the number of confirmed cases of the virus as of August 2021 [6]. Geriatric mental health has often been given a pass, and the difficulties of the elderly are dismissed as part of the normal aging process. In China, it was seen that the elderly population had lower levels of awareness and scanty access to error-free information about the outbreak of the pandemic which resulted in either excessive worrying or disregard to warnings [7]. Many who lived alone or did not have caregivers lost even their domestic help due to travel prohibitions or quarantine requirements [7]. Loneliness is an actual risk factor to health and well-being, and elderly people are vulnerable to it even in the usual times, as social circles shrink with age due to declining and chronic health conditions, death of partners and friends, and thus they may have fewer closer relationships and more likely to live alone [3, 8]. Imposed social isolation aggravates the loneliness of older people and disproportionately limits the daily interactions that would otherwise occur during grocery shopping, attending community activities, or at places of worship [3].

Change in work structure, employment opportunities, and demographics have now left several elderly people to live by themselves while their children and grandchildren earn and reside in other cities or countries. This acute crisis holds important lessons for our understanding of social isolation, dynamics of societal ties in times of strain, loneliness, and how to formulate cost-effective management for the debilitating effects of the pandemic. The biological and psychosocial resilience manifested by the elderly additionally requires to be understood as a protective and preventive factor [4].

The fear of contracting the disease and losing loved ones is mental stress and anxiety which may perplex the already grim situation. The Ebola outbreak in Africa during 2014–2016 saw many people being affected by mental health issues [9]. Recent studies had pointed out a prevalence of 10.8% and 62.16% of anxiety disorders and depression in the elderly population in community-based studies [10, 11].

The Government of India has also identified geriatric and pediatric mental health issues among its priority during the COVID-19 pandemic. Through the National Institute of Mental Health and Neurosciences (NIMHANS), messages and educational material have been circulated which emphasizes points of spending time with family, spending more time on recreational activities during COVID-19, making some time for exercise like stretching, yoga, and walking indoors, connecting to loved ones near and dear, and cutting down on listening to grim news. These messages should be practiced along with advisory given by the government on handwashing and cough etiquettes [12]. The present study was conceived to see whether the COVID-19 pandemic causes anxiety and depression in the elderly.

## Methods

### Participants

The current sample consisted of those who were aged 60 years and above, residing in two sectors of Chandigarh city which were randomly selected (sectors 61 and 37). Samples were randomly selected using a computer-generated random table. The inclusion criteria comprised those who were over 60 years and residing in Chandigarh for the past 1 year, who were willing to give consent, and whose Everyday Abilities Scale for India (EASI) score did not exceed “zero.” This was done to exclude any individuals with a cognitive impairment like dementia and EASI can be used on caregivers/family members as an alternative to the instruments like Hindi Mental State Examination (HMSE) [13]. Those found to be bedridden due to some severe physical illness, having difficulty in speaking and/or hearing, or receiving treatment for any psychiatric disorder in the past year were excluded from the study.

The sample size was calculated by taking prevalence of depression as 62.16% [11], precision as 5%, and 95% confidence interval. With a finite population size of 122 geriatric individuals residing in the study area, a sample of 92 individuals above the age of 60 years was calculated.

A total of 112 participants, in the range of 60 to 86 years, were telephonically contacted. Three of them did not give consent while three calls were not received (over three attempts made at the interval of 24 h). One record was excluded because the participant reported taking psychiatric consultation for depression over the past 1 year. Thirteen records were further excluded as their EASI score exceeded “zero.” Ninety-two participants were finally incorporated in the study. The data was collected over 45 days, spread over May and June 2020.

### Procedure

The study was initiated after finalizing the list of individuals to be approached for the study, based on a computer-generated random table. Those aged 60 and above were then telephonically contacted and consent was sought. Participants were briefly explained the context and purpose of the present study and were informed that they would be free to decline or withdraw from the study at any point in time in the research. They were also assured regarding the confidentiality of their responses. Once verbal consent was obtained, they were asked if they had been seeking any sort of psychiatric consultation over the past year. Those who denied were further asked about the basic sociodemographic details and requested if speaking with their caregiver would be permitted. With their permission, their caregivers were approached and EASI was administered to know about the functional activity of the elderly resident as well as to screen for the presence of dementia-like illness. Those who scored “zero” on EASI were further included to administer the clinical datasheet and General Health Questionnaire-12 (GHQ-12) [14]. Those scoring > 2 on GHQ-12 were further administered the Geriatric Anxiety Inventory Hindi version (GAI) and Geriatric Depression Scale short version (GDS), and their responses were recorded. The average time taken for each call ranged between 15 and 20 min. Additionally, those scoring 1 or above on EASI or > 2 on GHQ were also guided to get in touch with the Department of Psychiatry for further evaluation.

The study was approved by the Institutional Ethics Committee and participants who had been found with psychiatric morbidity/illness were asked to undergo further evaluation.

### Measurements

Sociodemographic details including age, gender, caregiver whereabouts, marital status, education, occupation, family income per month, religion, residence, family type, languages known, and presence of any chronic illness—its nature, duration, and ongoing medications—were recorded.

The 12-item Everyday Abilities Scale for India (EASI- Hindi version as Hindi was local vernacular language) was administered to assess the participants’ current day-to-day functional abilities and dementia-like illness [15]. Each question is answered as a Yes/No leading to a score of “one” where the subject is found to be facing difficulties in carrying out that particular function while a score of “zero” indicates adequate functioning. Higher EASI scores denote higher dysfunction.

The 12-item General Health Questionnaire (GHQ-12) was administered to screen for the presence of any of the common mental disorders besides assessing the overall psychological and psychiatric well-being of the participants. Each item is scored as either “zero” or one with the former indicating absence or usual amounts of the mental problem. A score of GHQ > 2 indicates the presence of psychological morbidity [14].

The Geriatric Anxiety Inventory Hindi version (GAI-Hindi) [16] consisting of 20 items and Geriatric Depression Scale short version (GDS) [17] comprising 15 items was administered to assess the presence and severity of anxiety and depressive symptoms respectively among the elderly participants. The items of either scale are answered as a “Yes” or “No” with the former suggesting the presence of the symptom. A score of more than 8 on GAI-Hindi and 5 or more on GDS indicates the presence of anxiety and depression and needs evaluation [16, 17].

### Statistical analysis

The collected data were analyzed using the SYSTAT software for Windows (version 13.2). Quantitative data were presented in terms of mean and standard deviation while qualitative data were presented as frequency and proportions. Association between qualitative variables was analyzed using the chi-square test. The strength of association of sociodemographic factors with anxiety and depression was measured in terms of the odds ratio. The point of statistical significance was considered when the *p* value was found out to be less than 0.05 (*p* < 0.05).

## Results

From the list of 112 subjects, 20 subjects could not be included for administering the questionnaire for a variety of reasons leaving a final sample of 92 geriatric patients. Males outnumbered the females with a ratio of 1.8:1. Three fourth of the subjects were in the age category of 60–70 years. The distribution of religion was Hindu (70.7%), Sikh (26.1%), and others (3.3%). In the present study, 15.2% had some sort of psychiatric morbidity as assessed by GHQ-12.

Patients who had chronic illnesses showed a 4.5 times greater chance of having psychological morbidity (GHQ > 2) (Table 1). Half (50%) of the subjects had one or more comorbidity, the most common being hypertension (27.2%) followed by diabetes (21.7%). Other comorbidities included arthritis (5.4%), cardiac illness (4.3%), and asthma (2.2%). In the majority of subjects, co-morbidities were of more than 3 years duration (41/92, 44.6%).

**Table 1:** Demographic characteristics of the geriatric sample and univariate analysis with odds ratio (95% C.I) for psychiatric morbidity (GHQ > 2)

Variable	Categories	Frequency (%)	Odds ratio (95% C.I.)	***p*** value
Age	60–70 years	69 (75%)	Reference	0.102
	70 years and above	23 (25%)	2.691 (0.821–8.821)	
Gender	Male	59 (64.1%)	Reference	0.231
	Female	33 (35.9%	0.436 (0.112–1.693)	
Caregiver	Self	15 (16.3%)	Reference	
	Spouse	70 (76.1%)	3.19 (0.38–26.50)	0.282
	Children	7 (7.6%)	0.00	0.999
Marital status	Married	83 (90.2%)	Reference	
	Widowed	9 (9.8%)	0.673 (0.078–5.84)	0.720
Education	Below matric	20 (21.8%)	Reference	
	Intermediate	11 (12.0%)	2.125 (0.349–12.95)	0.414
	Graduate	50 (54.3%)	0.493 (0.10–2.43)	0.385
	Professional	11 (12.0%)	3.238 (0.57–18.38)	0.185
Occupation	Professional/own business	7 (7.6%)	Reference	
	Retired	66 (71.7%)	0.211 (0.040–1.10)	0.065
	Homemaker	19 (20.7%)	0.157 (0.019–1.274)	0.083
Family income	10 k–20 k	26 (28.3%)	Reference	
	20 k–30 k	43 (46.7%)	2.333 (0.446–12.20)	0.315
	> 30 k	23 (25%)	3.333 (0.579–19.18)	0.178
Family type	Nuclear	81 (88%)	0.783 (0.150–4.076)	0.771
	Extended/joint	11 (12%)	Reference	
Chronic illness	Present	46 (50%)	4.505 (1.165–17.42)	0.029*
	Absent	46 (50%)	Reference	

*Statistically significant (*p* < 0.05)

On Geriatric Anxiety Scale, scores of more than 8 were present in 8/92 (8.7%) subjects whereas a score of 5 or more in GDS-15 was seen in 14/92 (15.2%) subjects. There was a poor correlation between GAI and GDS (*r* = 0.314, *p* value = 0.275).

## Discussion

It is now widely known that elderly people and those with existing conditions like diabetes and hypertension are at high risk to develop severe COVID-19 infections; especially, the fatality is relatively more in the elderly [18]. News like these get repeated quite often, which only adds to the anxiety of this group of the vulnerable population. Anticipating this, steps like social isolation, use of digital technology to take care of some of the needs (like banking and commerce), help through non-governmental organizations, and telephone helplines have come into place to mitigate some of the problems of the elderly [19]. However, the resilience of the elderly during these trying times has also been reported, with studies pointing out that the elderly somehow have a better mental health outcome in comparison to the younger population [20]. The present study explored the prevalence of anxiety and depression among the elderly population in an urban region due to COVID-19.

Among the study participants, 75% of the participants were in the age group of 60–70 years, and majority of the participants were male and were married; most of them were in a nuclear family, were retired, and had a graduation degree. Family income was in the range of rupees 20,000–30,000 per month in about 47% of the participants. Univariate analysis done in the study population which was divided into various sub-groups did not reveal any significant difference among them in terms of developing psychological distress.

Half of the study population also had chronic illnesses like diabetes and hypertension. Univariate analysis showed that those who had comorbid physical illness had 4.5 times the risk of having psychological problems. This is quite understandable as people with physical illness are vulnerable to have mental illness [21], and this stands true for the elderly population as well. The severity of COVID-19 in the elderly and those with chronic illnesses like hypertension and diabetes is more [18], and the knowledgeable elderly population is aware of this. So this itself may result in apprehension, stress, and depressive cognition in the elderly population of this study.

In an online survey done among the elderly in Greece, moderate to severe depressive symptoms (81.6%), anxiety (84.5%), and disrupted sleep (37.9%) were reported [22]. Another study conducted pan India in 64 cities found mild and moderate psychological impact in 15.0% and 5.5% respectively. The severe psychological impact was reported in 12.7% of subjects [23]. This study also found that higher age was associated with lesser psychological impact. The current study found psychiatric morbidity of 15.2% which is comparable to the above study. A lower prevalence among the elderly could be due to higher resilience and coping mechanism as already stated in previous studies [20]^.^

Our study had shown that with the presence of chronic illnesses such as hypertension and diabetes, there were significantly higher odds of psychiatric morbidity. Varshney et al. also had a similar finding from their online study of 653 subjects [23]. The presence of physical morbidity such as diabetes, smoking, hypertension, frailty, and obesity has already been associated with higher mortality among COVID-19 patients which may explain the higher odds of psychiatric morbidity also.

Regarding anxiety among geriatric subjects, a study in Turkey found severe anxiety levels regarding individuals in their families (14.1%), their social life (10.1%), and their economic status (19.7%) [24]. A study done in Kerala state using GAS-30 found 37.9% and 22.6% anxiety levels using cutoff levels of 8/9 and 16 [25]. Another study done in Iran had found higher levels of anxiety in the geriatric population with 60% and 46.6% reported symptoms of anxiety and depression on the GAS-10 and GDS-15 scale [26]. Lower levels of anxiety in the current study could be explained through protective factors such as the presence of caregiver support (spouse and children, 83.7%), being married (90.2%), good education level (> 2/3rd graduate/professional), and an income > 20,000 INR (71.7%) with depressive symptoms present in 15.2% of the geriatric population in the current study.

The study had some limitations which included a small sample size. Other limitations included response bias as this was a telephonic data collection. Also, the study was questionnaire-based and reasons for anxiety and depression were not enquired.

## Conclusions

The present study found lower levels of anxiety and depressive symptoms among the geriatric population due to COVID-19 as compared to populations in other parts of the world which may be attributable to higher resilience. The presence of co-morbidity was significantly associated with psychological morbidity. More studies may be conducted to reflect the psychological situation of the geriatric population to validate the findings of the study.

## Data Availability

All the data and materials relevant to this study are available with the corresponding author, and anonymized data can be made available at a reasonable request.

## Abbreviations

COVID-19: Coronavirus disease 2019
EASI: Everyday abilities scale for India
GAI: Geriatric Anxiety Inventory
GDS: Geriatric Depression Scale short version
GHQ-12: General Health Questionnaire 12
HMS: Hindi Mental State Examination
NIMHANS: National Institute of Mental Health and Neurosciences
